# Matched comparative study of trifocal bone transport versus induced membrane followed by trifocal bone transport in the treatment of segmental tibial defects caused by posttraumatic osteomyelitis

**DOI:** 10.1186/s12891-022-05501-8

**Published:** 2022-06-14

**Authors:** Yimurang Hamiti, Maimaiaili Yushan, Ainizier Yalikun, Cheng Lu, Aihemaitijiang Yusufu

**Affiliations:** Department of Microrepair and Reconstructive Surgery, The First Affiliated Hospital of Xinjiang Medical University, Urumqi, Xinjiang People’s Republic of China

**Keywords:** External fixator, Induced membrane, Ilizarov technique, Trifocal bone transport, Osteomyelitis

## Abstract

**Objectives:**

To compare the efficacy and clinical outcomes of trifocal bone transport (TBT) versus induced membrane followed by trifocal bone transport (IM + TBT) in the treatment of tibial defects **>** 6 cm caused by posttraumatic osteomyelitis.

**Methods:**

A total of 69 eligible patients with tibial defects > 6 cm who were treated between January 2010 and January 2018 were retrospectively reviewed. Overall, 18 patients treated by IM + TBT and 18 treated by TBT were matched by propensity score analysis. The mean tibial defect after radical debridement was 6.97 ± 0.76 cm (range, 6.0 to 8.9 cm). The measurements, including demographic data, external fixation index (EFI), external fixation time (EFT), duration of docking union, bone and functional outcomes evaluated by the Association for the Study and Application of the Method of Ilizarov (ASAMI) scoring system, and postoperative complications evaluated by Paley classification during follow-up were recorded.

**Results:**

Age, gender, injury mechanism, affected side, defect size, previous operation time, and follow-up time were not significantly different between the two groups (*P* > 0.05). The mean EFT was 293.8 ± 12.1 days in the TBT group vs. 287.5 ± 15.3 days in the IM + TBT group. The mean EFI was 36.02 ± 2.76 days/cm vs. 34.69 ± 2.83 days/cm, respectively. The mean duration of docking union was 210.7 ± 33.6 days vs. 179.7 ± 22.9 days, respectively. There was no significant difference in postoperative bone and functional results between the two groups. Delayed union or nonunion and soft tissue incarceration were significantly reduced in the IM + TBT group compared to those in the TBT group.

**Conclusion:**

Both TBT and IM + TBT achieved satisfactory postoperative bone and functional outcomes in patients with segmental tibial defects > 6 cm following posttraumatic osteomyelitis, while IM + TBT had a significantly lower incidence of postoperative complication in delayed union or nonunion and soft tissue incarceration, as well as faster docking union.

**Supplementary Information:**

The online version contains supplementary material available at 10.1186/s12891-022-05501-8.

## Introduction

Segmental tibial defects caused by posttraumatic osteomyelitis are a common clinical condition, and management continues to be a major challenge for surgeons, especially when infection or soft tissue loss compromises the surrounding blood supply for bone regeneration [1–3]. Several reconstructive techniques are available, including the induced membrane technique, Ilizarov bone transport technique, and autologous and allografts bone grafting [1–12]. Although the aforementioned therapeutic approaches have several advantages and disadvantages, it is essential to modify repair procedures for each patient to obtain the desired clinical outcome.

The induced membrane technique was described by Masquelet in the 1980s and has been considered to be the main method of treatment for segmental bone defects. It is a two-stage process; first, debridement is performed and the bone defect site is filled with a polymethylmethacrylate (PMMA) spacer, which can induce the formation of a biological membrane in the first stage, then the defect space is reconstructed following cement removal in the second stage [3, 9]. Similarly, the Ilizarov bone transport technique has been advocated as a viable surgical approach for the treatment of segmental tibial defects following posttraumatic osteomyelitis. Studies such as trifocal or multifocal bone transport have been conducted to reduce the time required for external fixation using the classic Ilizarov procedure and its related disadvantages [5, 13–19]. The combined use of induced membrane and bifocal bone transport has demonstrated its effectiveness in successfully reconstructing bone defects [20–24].

The clinical outcomes of 36 patients who underwent surgery with TBT or IM + TBT for segmental tibial defects greater than 6 cm caused by posttraumatic osteomyelitis were examined in this research. Propensity score match (PSM) analysis was used to compare these different reconstructive strategies in terms of postoperative outcomes of the patients. To the best of our knowledge, this is the first paper that compares the efficacy and clinical outcomes of TBT and IM + TBT in this field.

## Patients and methods

### Patients

This was a single-center retrospective comparative study. Preoperative planning and X-ray evaluation were performed by the senior authors. All surgeries were performed by members of the same surgical team. The study protocol was approved by the Institutional Ethics Committee of our institute, which waived the requirement for obtaining informed consent from the participants. Patients with tibial defects **>** 6 cm (after radical debridement) following posttraumatic osteomyelitis who were treated by TBT or IM + TBT between June 2010 and June 2018 were retrospectively reviewed. Patients with tibial defects due to other causes, such as tumor removal, were < 18 years of age, or had inadequate follow-up data were excluded from the study. Overall, 69 patients were included in the present study. Among these, 51 patients underwent trifocal bone transport, and 18 patients received the application of the induced membrane in the first stage followed by trifocal bone transport in the second stage. The included patients underwent a minimum follow-up of 2 years after the external fixator was removed. Data, including radiological findings, operation records, and medical history, were retrieved from medical records and analyzed by three surgeons (MY, CL and AY). Demographic and baseline information, such as age, gender, injury mechanism, affected side, defect size and location, previous operation time, and follow-up time, was recorded by two surgeons (YH and AYu).

### Matched variables

Given the potential differences in the patients’ demographic and baseline data between the TBT and IM + TBT groups, we performed a propensity score match analysis to compare the intergroup differences. The primary and additional outcomes might be compared concurrently based on comparable baseline features. Demographic and baseline data, such as age, gender, injury mechanism, affected side, defect size, previous operation time, and follow-up period, were all collected as variables.

### Outcome evaluation

All patients who participated in the study were followed up regularly. Outpatient follow-up was performed every two weeks and consisted of radiographic examination until radiographic evidence of union and pain-free mobilization were observed. The rate and rhythm were changed based on radiographic assessments of each distracted region and the patient’s endurance. The external fixator was removed after one month of dynamization when the transferred segment arrived at the docking site and at least three bridging callus appeared on anteroposterior and lateral radiographs [25].

The external fixation time (EFT) was defined as the period from when the frame was applied to when it was removed (in days). The external fixation index (EFI) was recorded by dividing the EFT by the length of the regenerated bone (in days/cm). The total length of time (in days) after the completion of distraction necessary to the removal of external fixation to produce the indications of the union at the docking site was described as the duration of the docking union. Assessment of bone and functional outcomes used the Association for the Study and Application of the Method of Ilizarov (ASAMI) score system [5]. Complications that occurred during the treatment period were recorded and graded according to the Paley classification as problems, obstacles, and true complications [26]. This classification system was used to standardize the evaluation of adverse events associated with deformity repair and lengthening procedures. A problem was characterized as a possible difficulty that developed throughout therapy but did not necessitate surgical intervention and was entirely addressed at the end of treatment. An obstacle was an adverse event during treatment that was totally resolved by the conclusion of the treatment period by operational intervention. A true complication was an issue that persisted after the therapy time ended.

### Statistical analysis

Statistical analysis was performed using SPSS 25.0 software (SPSS software, Chicago, IL, USA). The obtained data were first tested for normal distribution. Continuous variables are presented as the mean ± standard deviation while proportions are presented for categorical variables. Student’s t-tests or Mann-Whitney U-tests were performed to compare the differences between the two groups as appropriate. Categorical data were evaluated with Fisher’s exact test. A *P*-value of **<** 0.05 indicated a statistically significant difference.

## Results

### General results

**Fig. 1:**
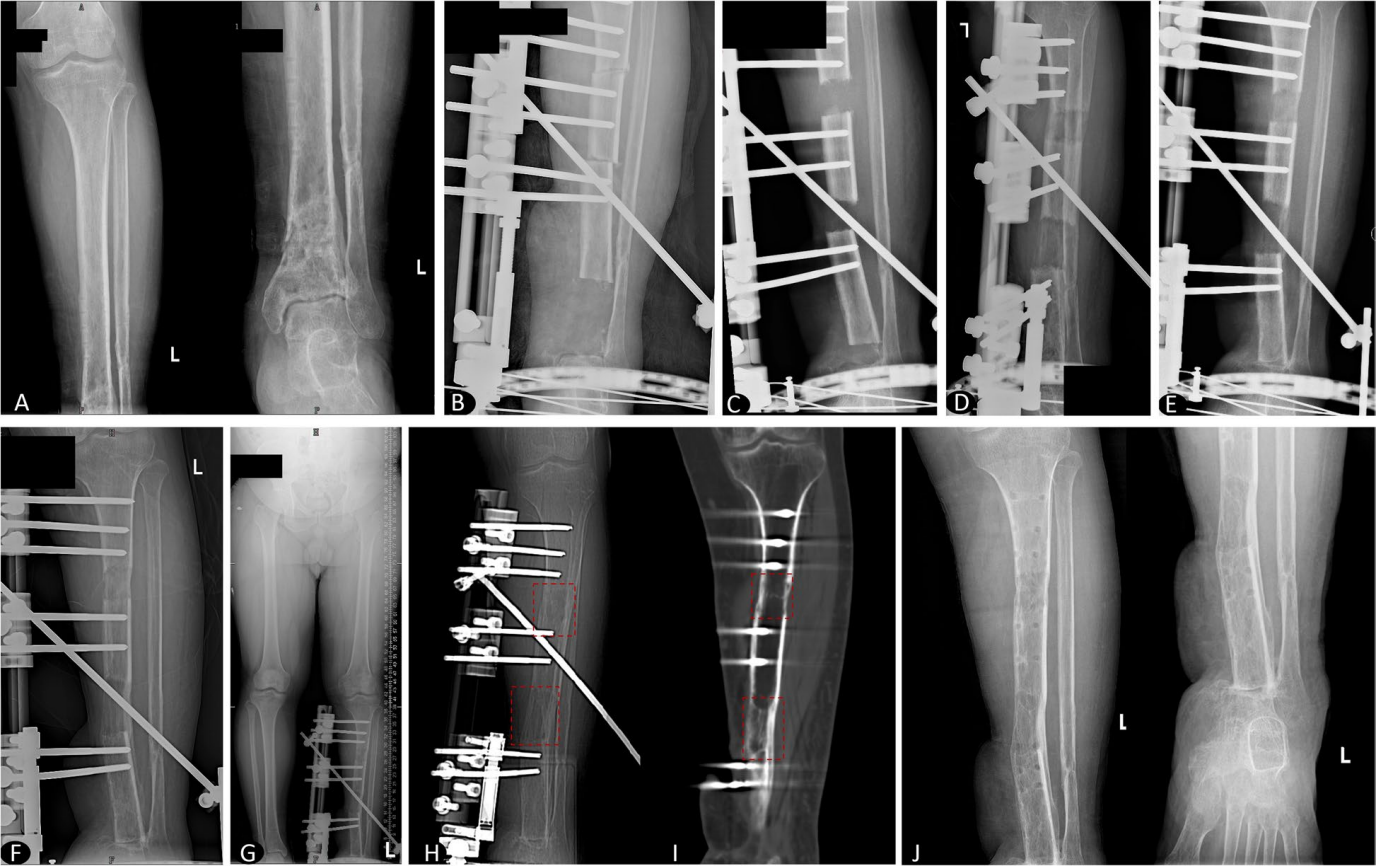
**A** A 28-year-old male patient with postoperative infection in open tibial fractures. **B** An excision of infected bone and soft tissue with 7.3 cm defect and beginning of trifocal bone transport. **C** One month after trifocal bone transport. **D, E, F** 1 month, 3 months, 6 months after the docking was reached. **G, H, I** Trifocal bone transport was completed with good regenerate consolidation, and docking union was achieved and evaluated on X-ray and CT. **J** Radiograph appearance with an excellent bone result at 2 years after the removal of the external fixator

**Fig. 2:**
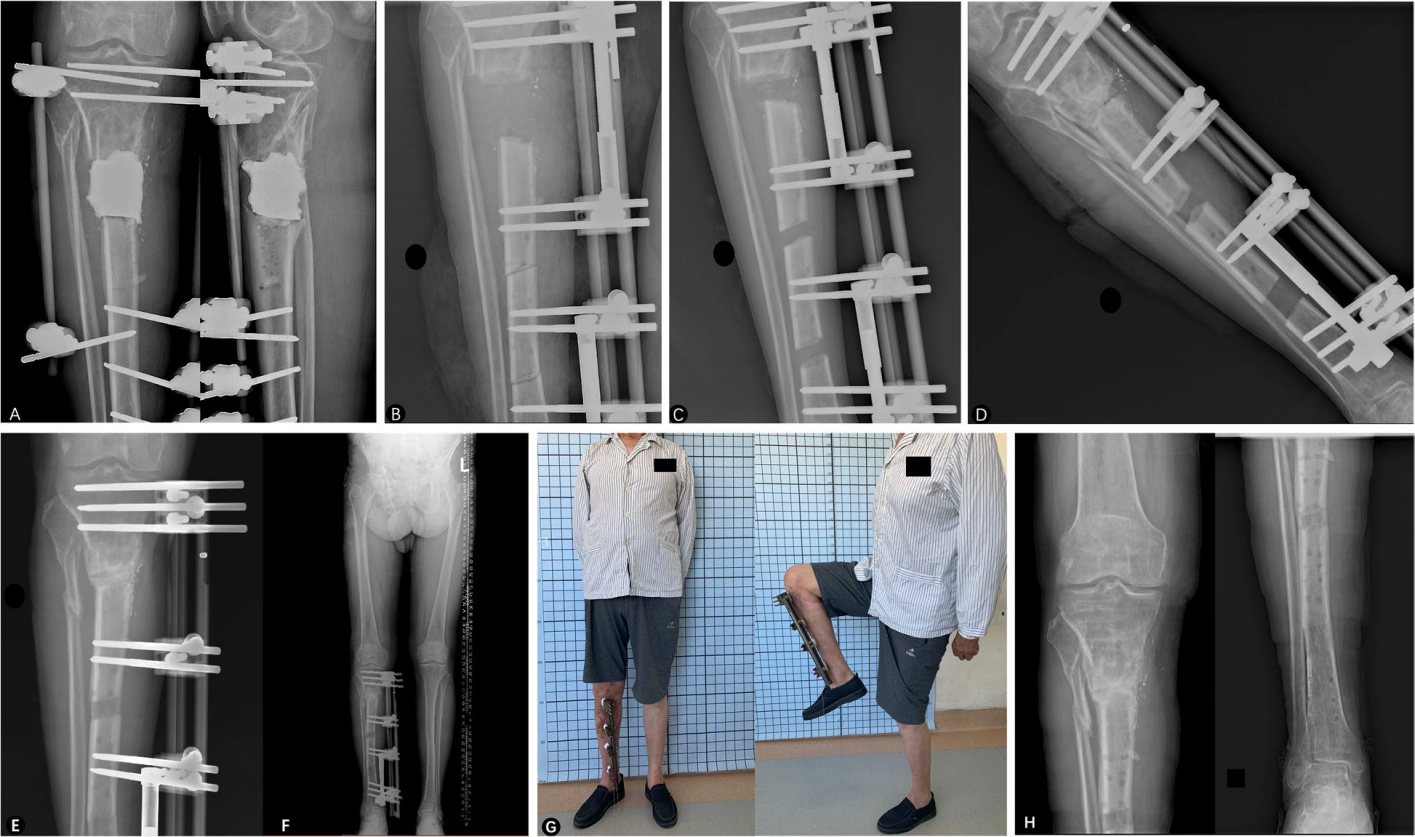
**A** A 53-year-old male patient with posttraumatic osteomyelitis of the right tibia. After previous debridement operations, there was a defect of 6.1 cm and filled with PMMA spacer. **B,C** The PMMA spacer was removed 4 weeks later and the trifocal bone transport was started. **D** Docking was reached at 42 days after bone transport on X-ray. **E,F** Satisfied regenerates on both distracted area and docking union were achieved 92 days the docking contact. **G** General appearance before the removal of the external fixator with excellent functional result. **H** Radiograph appearance with an excellent bone result at 26 months after the removal of external fixator

A total of 36 patients, including 18 patients in the IM + TBT group and 18 of the 51 patients in the TBT group, were entered into the present study after 1:1 propensity score matching. The demographic and preoperative baseline data of the two groups are provided in Table 1. All patients underwent a minimum of two years of follow-up with an average of 27 months. The mean age of the patients was 41.3 ± 10.7 years (range, 21 to 62 years). The injury mechanism included transport accidents in 18 patients, falls in 6, and crushes in 12. The average length of tibial defects after radical debridement was 6.97 ± 0.76 cm (range, 6.0 to 8.9 cm). There was no significant difference in demographic or baseline data between the groups (*P* > 0.05). Typical cases are shown in Fig. 1 and Fig. 2.

**Table 1:** Comparison of the demographic and preoperative baseline data

Parameter	Total	TBT group	IM + TBT group	*P*-value
Mean age (years)	41.3 ± 10.7	41.4 ± 10.9	41.1 ± 10.8	0.915
Gender (male/female)	26/10	12/6	14/4	0.711
Injury mechanism (transport accidents/falls/crushes)	18/6/12	10/3/5	8/3/7	0.467
Affected side (left/right)	20/16	11/7	9/9	0.738
Mean defect size (cm)	6.97 ± 0.76	6.89 ± 0.74	7.05 ± 0.79	0.546
Median previous operation time (n, IQR)	2 (1 to 3)	2 (1 to 3)	2 (1.75 to 3)	0.249
Median follow-up period (months, IQR)	27 (25 to 37.6)	29 (25.8 to 38)	26.5 (25 to 32)	0.301

*TBT* trifocal bone transport, *IM + TBT* induced membrane followed by trifocal bone transport, *IQR* interquartile range

### Postoperative outcomes

The comparison of postoperative data is shown in Table 2. The mean EFT was 293.8 ± 12.1 days (range, 280 to 320 days) in the TBT group and 287.5 ± 15.3 days (range, 266 to 311 days) in the IM + TBT group. The mean EFI was 36.02 ± 2.76 days/cm (range, 30.8 to 40.6 days/cm) in the TBT group, and 34.69 ± 2.83 days/cm (range, 30.1 to 40.2 days/cm) in the IM + TBT group. The EFT and EFI were shorter in the IM + TBT group than in the TBT group. However, there were no statistically significant differences between the two observed results. The mean duration of docking union was 210.7 ± 33.6 days (range, 162 to 260 days) in the TBT group and 179.7 ± 22.9 days (range, 144 to 212 days) in the IM + TBT group. There was a significant difference between the two groups (*P* < 0.05).

**Table 2:** Comparison of the postoperative outcomes

	TBT group	IM + TBT group	*P*-value
Mean EFT (days)	293.8 ± 12.1	287.5 ± 15.3	0.177
Mean EFI (days/cm)	36.02 ± 2.76	34.69 ± 2.83	0.163
Duration of docking union (days)	210.7 ± 33.6	179.7 ± 22.9	0.003

*TBT* trifocal bone transport, *IM + TBT* induced membrane followed by trifocal bone transport, *EFT* external fixation time, *EFI* external fixation index

The Association for the Study and Application of the Ilizarov Method (ASAMI) scoring system was used to evaluate bone and functional outcomes [5], which are summarized in Table 3. In the TBT group, the bone outcomes were excellent in 7 patients (38.9%), good in 7 (38.9%), fair in 3 (16.7%), and poor in 1 (5.6%). The functional outcomes were excellent in 9 patients (50.0%), good in 6 (33.3%), fair in 2 (11.1%), and poor in 1 (5.6%). In the IM + TBT group, the bone outcomes were excellent in 5 patients (27.8%), good in 8 (44.4%), fair in 3 (16.7%), and poor in 2 (11.1%). The functional outcomes were excellent in 4 patients (22.2%), good in 9 (50.0%), fair in 3 (16.7%), and poor in 2 (11.1%). With no significant differences between the two groups, both achieved satisfactory bone and functional outcomes.

**Table 3:** Comparison of the bone and functional results according ASAMI classification

Outcomes	Treatment	Numbers/Percentage				*P*-value
		Excellent	Good	Fair	Poor	
Bone results	TBT	7 (38.9%)	7 (38.9%)	3 (16.7%)	1 (5.6%)	0.480
	IM + TBT	5 (27.8%)	8 (44.4%)	3 (16.7%)	2(11.1%)	
Functional results	TBT	9 (50.0%)	6 (33.3%)	2 (11.1%)	1 (5.6%)	0.113
	IM + TBT	4 (22.2%)	9 (50.0%)	3 (16.7%)	2(11.1%)	

*TBT* trifocal bone transport, *IM + TBT* induced membrane followed by trifocal bone transport

The complications shown in Table 4 were evaluated using the standards proposed by Paley [25]. In the TBT group, there were 57 complications, including 26 problems, 21 obstacles, and 10 true complications. The complication rates were 66.7% for muscle contraction, 38.9% for soft tissue incarceration, 38.9% for axial deviation, 72.2% for pin problems, 16.7% for delayed consolidation, 50.0% for delayed union or nonunion, and 27.8% for joint stiffness. In the IM + TBT group, there were 47 complications, including 26 problems, 9 obstacles, and 12 true complications. The complication rates were 44.5% for muscle contraction, zero for soft tissue incarceration, 44.5% for axial deviation, 83.3% for pin problems, 33.3% for delayed consolidation, 11.1% for delayed union or nonunion, and 44.5% for joint stiffness. The postoperative complication rate in delayed union or nonunion and soft tissue incarceration differed significantly among the groups.

**Table 4:** Comparison of the complications according Paley criteria

Parameter	Treatment	Problems	Obstacles	True Complications	Total	*P*-value
Muscle contraction	TBT	7	2	3	12	0.315
	IM + TBT	5	0	3	8	
Soft tissue incarceration	TBT	0	6	1	7	0.008
	IM + TBT	0	0	0	0	
Axial deviation	TBT	4	1	2	7	0.730
	IM + TBT	1	3	4	8	
Pin problems	TBT	9	4	0	13	0.691
	IM + TBT	12	3	0	15	
Delayed consolidation	TBT	3	0	0	3	0.443
	IM + TBT	5	1	0	6	
Delayed union or nonunion	TBT	2	7	0	9	0.027
	IM + TBT	0	2	0	2	
Joint stiffness	TBT	1	1	3	5	0.489
	IM + TBT	3	0	5	8	
Other	TBT	0	0	1	1	1.000
	IM + TBT	0	0	0	0	
Total		52	30	22		

*TBT* trifocal bone transport, *IM + TBT* induced membrane followed by trifocal bone transport

## Discussion

The most important finding of this study was that patients with tibial defects greater than 6 cm caused by posttraumatic osteomyelitis who were treated with IM + TBT had significantly lower postoperative complication rates in delayed union or nonunion and soft tissue incarceration, as well as faster docking union than those treated with TBT. Both the TBT and IM + TBT achieved satisfactory bone reconstruction outcomes with no significant differences in bone and functional outcomes between the two groups. TBT and IM + TBT had similar results regarding the treatment of segmental tibial bone defects (> 6 cm) caused by posttraumatic osteomyelitis.

The management of large bone defects due to posttraumatic osteomyelitis of the tibia is extremely challenging. Treatment strategies include complete debridement of the infected area to control the inflammation and reconstruction of the bone and soft tissue defect. The Ilizarov bone transport technique and the induced membrane technique are more traditional reconstructive techniques that have been commonly applied to treat segmental tibial bone defects [1, 3, 4].

The long period required for external fixation increases external fixation-related problems such as pin tract infection and becomes the primary impediment to the Ilizarov bone transport technique [5, 13–19]. To minimize the unavoidable long period of treatment using the classic Ilizarov procedure and its related disadvantages, trifocal bone transport (TBT) was proposed to decrease the duration of treatment. Paley et al. [13] presented the treatment of 19 patients with tibial defects by using single- or double-level bone transport. The mean duration of EFT was 16 months. The mean EFI was 2.1 months/cm and 1.2 months/cm, respectively. Zhang et al. [14] reported 16 patients who had mean tibial defects of 10.9 ± 3.8 cm following radical resection and were treated with double-level bone transport. The mean EFT and EFI were 12.0 ± 3.9 months and 1.1 ± 0.3 months/cm, respectively. Xu et al. [15] published a series of 31 patients with massive tibial bone and soft tissue defects who were treated with the trifocal bone transport technique. The mean bone defect was 11.87 ± 2.78 cm (range, 8.2 to 18.2 cm). The mean EFT was 22.74 ± 6.82 months, and the mean EFI was 1.91 ± 0.3 months/cm. Trifocal and bifocal bone transport were compared in the treatment of long tibial bone defects by Catagni et al. [16], indicating that trifocal bone transport can greatly minimize the time required for tibial repair and reduce the need for additional surgeries and associated complications. Another comparative study presented by Yushan et al. [17] demonstrated that when trifocal bone transport is used instead of bifocal bone transport, the length of regeneration consolidation and docking union can be significantly reduced with superior functional outcomes. Before performing the multilevel bone transport, several caveats should be considered. Blood supply is crucial for enhancing osteogenesis and bone regeneration, and the nutrient foramina of the tibia are in the proximal third of its diaphysis [27]. Hence, the appropriate osteotomy level should be chosen to avoid tibial nutrient foramina. Osteotomy is commonly performed in the metaphysis of the tibia because of its abundant blood supply and substantial bone surface area. There is an ongoing debate about whether soft tissue defects should be covered up early in the process of treatment. Early soft-tissue coverings were recommended by Spiegl et al. [21] to limit dead space, restore adequate local blood flow, and raise local antibiotic concentrations. However, other authors presented different opinions of the treatment. As a result, based on established evidence, it was problematic to offer any definitive recommendations for clinical practice. In our opinion, using a flap at an early stage in the reconstruction of composite bone and soft tissue loss may help to minimize the risk of reinfection and the frequency of dressing changes; most importantly, sufficient soft tissue coverage is crucial to docking union, which in turn reduces discomfort and financial burden on the patient.

The classic Masquelet technique was separated into two stages, which involve a complete debridement of bone and soft tissues in the first stage, followed by the placement of PMMA cement in the bone defect area and removal of the cement in the second stage to repair the defect according to the mechanical and biological properties of the induced membrane. The conventional induced membrane technique involves the application of autologous or allogeneic bone grafts to fill bone defects after cement removal, which has a high healing rate, in the second stage, but its shortcomings include bone resorption, refracture, deformity, etc. [1, 3, 9].

In the IM + TBT group, TBT was performed instead of autologous or allogeneic bone grafting after the removal of the PMMA spacer in the second stage. Several studies with a similar methodology to the presented research were proposed. Marais et al. [20] described the use of the Ilizarov bone transport technique through an induced membrane to treat 7 patients with tibial bone defects. The mean EFT was 77 weeks, and the mean EFI was 81 days/cm. Spiegl et al. [21] observed an average EFI of 57 days/cm in a group of patients with chronic osteomyelitis. A comparative study conducted by van Niekerk et al. [22] revealed that antibiotic-impregnated spacers for open tibial trauma were beneficial and reduced the EFI by a significant amount. Antibiotics in the cement spacer are still a point of contention. Several previous articles indicated that antibiotics mixed with cement would be slowly released after several weeks of placement in the defect area. Bacterial growth is prevented and a sterile environment is created by a 200-fold increase in local antibiotic concentrations [20–24, 28, 29]. Another study found that antibiotics may disguise the effect of insufficient debridement by reducing but not eliminating any subsequent infection [30]. In the present study, one case of osteomyelitis recurred in the TBT group, but none recurred in the IM + TBT group. The fact that the initial stage of surgical treatment was not performed radically may account for some of this.

In comparison to the classic Ilizarov bone transport process, PMMA spacers may provide some benefits in a reconstructed postinfection environment. The placement of cement prevented the incarceration of soft tissue and induced a body reaction that resulted in the construction of a biological membrane that served as a conduit for the bone segment to be transported in a stable environment following the removal of the cement spacer [20–24]. The biological membrane, also described as the induced membrane, is primarily composed of type I collagen and is a rich source of mesenchymal stem cells with mature vascularized fibrous tissue. Previous studies have proven the effect of induced membrane on osteogenesis enhancement and confirmed that the structures and abilities were similar to those of the periosteum [1, 3, 9, 31]. The membrane has a high concentration of growth factors that can secrete a number of osteogenic and neovascularizing growth factors, such as bone morphogenetic protein 2 (BMP-2) and vascular endothelial growth factor (VEGF). Several studies have been conducted and discussed the application of growth factors and indicated that they have the potential to significantly accelerate osteogenesis, bone regeneration, and rapid osseointegration [32–34].

Pin site infection was the most common complication in this trial, occurring in 13 patients in the TBT group and 15 patients in the IM + TBT group, which is consistent with the literature, and all resolved with improved pin tract care and oral antibiotic treatment based on the bacterial culture. In the reconstruction of tibial defects greater than 6 cm, the bone ends are often covered with a fibrocartilaginous cap, and the medullary canal is occluded at the time of docking contact. Soft tissue may become incarcerated between the bone ends, which is one of the important reasons for docking site delayed union or nonunion [35]. It is worth noting that the results showed significant decreases in the incidence of delayed docking union or nonunion, and soft tissue incarceration in the IM + TBT group compared with the TBT group. According to the above discussion, it could conceivably be hypothesized that the use of PMMA specaer and the formation of the induced membrane may reduce a formation of fibrocartilaginous cap and prevent soft tissue incarceration. However, given the small sample size, inferences should be interpreted with caution.

The present study had an important limitation. The current study is a retrospective comparative study conducted in a single center with a limited number of eligible clinical samples. Although the propensity score match analysis was performed to match variables. Large cohort prospective validation studies with a long follow-up period and large sample are needed in the future for a better assessment of the clinical validity of this method.

## Conclusion

In conclusion, the findings of this study have some implications for future clinical practice. Both TBT and IM + TBT achieved satisfactory postoperative bone and functional outcomes in the treatment of segmental tibial defects (> 6 cm) caused by posttraumatic osteomyelitis. Patients treated with IM + TBT had a significantly lower postoperative complication rate in terms of delayed union or nonunion and soft tissue incarceration, as well as faster docking union than those treated with TBT.

## Supplementary Information

**Additional file 1.**

## Data Availability

All data generated or analyzed during this study are included in this published article.

## References

[1] Borzunov DY, Kolchin SN, Malkova TA (2020). Role of the Ilizarov non-free bone plasty in the management of long bone defects and nonunion: problems solved and unsolved. World J Orthop.

[2] McNally M, Ferguson J, Kugan R, Stubbs D (2017). Ilizarov treatment protocols in the Management of Infected Nonunion of the tibia. J Orthop Trauma.

[3] Fung B, Hoit G, Schemitsch E, Godbout C, Nauth A (2020). The induced membrane technique for the management of long bone defects. Bone Joint J..

[4] Aktuglu K, Erol K, Vahabi A (2019). Ilizarov bone transport and treatment of critical-sized tibial bone defects: a narrative review. J Orthop Traumatol.

[5] Borzunov DY (2012). Long bone reconstruction using multilevel lengthening of bone defect fragments. Int Orthop.

[6] Aktuglu K, Günay H, Alakbarov J (2016). Monofocal bone transport technique for bone defects greater than 5 cm in tibia: our experience in a case series of 24 patients. Injury.

[7] Tetsworth K, Paley D, Sen C, Jaffe M, Maar DC, Glatt V (2017). Bone transport versus acute shortening for the management of infected tibial non-unions with bone defects. Injury.

[8] Oh CW, Apivatthakakul T, Oh JK, Kim JW, Lee HJ, Kyung HS (2013). Bone transport with an external fixator and a locking plate for segmental tibial defects. Bone Joint J..

[9] Morris R, Hossain M, Evans A, Pallister I (2017). Induced membrane technique for treating tibial defects gives mixed results. Bone Joint J.

[10] Ronga M, Cherubino M, Corona K, Fagetti A, Bertani B, Valdatta L (2019). Induced membrane technique for the treatment of severe acute tibial bone loss: preliminary experience at medium-term follow-up. Int Orthop.

[11] Meselhy MA, Singer MS, Halawa AM, Hosny GA, Adawy AH, Essawy OM (2018). Gradual fibular transfer by ilizarov external fixator in post-traumatic and post-infection large tibial bone defects. Arch Orthop Trauma Surg.

[12] Azzam W, Atef A (2016). Our experience in the management of segmental bone defects caused by gunshots. Int Orthop.

[13] Paley D, Maar DC (2000). Ilizarov bone transport treatment for tibial defects. J Orthop Trauma.

[14] Zhang Y, Wang Y, Di J, Peng A (2018). Double-level bone transport for large post-traumatic tibial bone defects: a single Centre experience of sixteen cases. Int Orthop.

[15] Xu YQ, Fan XY, He XQ, Wen HJ (2021). Reconstruction of massive tibial bone and soft tissue defects by trifocal bone transport combined with soft tissue distraction: experience from 31 cases. BMC Musculoskelet Disord.

[16] Catagni MA, Azzam W, Guerreschi F, Lovisetti L, Poli P, Khan MS (2019). Trifocal versus bifocal bone transport in treatment of long segmental tibial bone defects. Bone Joint J..

[17] Yushan M, Ren P, Abula A, Alike Y, Abulaiti A, Ma C (2020). Bifocal or trifocal (double-level) bone transport using unilateral rail system in the treatment of large tibial defects caused by infection: a retrospective study. Orthop Surg.

[18] Abuomira IE, Sala F, Elbatrawy Y, Lovisetti G, Alati S, Capitani D (2016). Distraction osteogenesis for tibial nonunion with bone loss using combined Ilizarov and Taylor spatial frames versus a conventional circular frame. Strategies Trauma Limb Reconstr.

[19] Li Y, Shen S, Xiao Q, Wang G, Yang H, Zhao H (2020). Efficacy comparison of double-level and single-level bone transport with Orthofix fixator for treatment of tibia fracture with massive bone defects. Int Orthop.

[20] Marais LC, Ferreira N (2015). Bone transport through an induced membrane in the management of tibial bone defects resulting from chronic osteomyelitis. Strategies Trauma Limb Reconstr..

[21] Spiegl U, Pätzold R, Friederichs J, Hungerer S, Militz M, Bühren V (2013). Clinical course, complication rate and outcome of segmental resection and distraction osteogenesis after chronic tibial osteitis. Injury..

[22] van Niekerk AH, Birkholtz FF, de Lange P, Tetsworth K, Hohmann E (2017). Circular external fixation and cemented PMMA spacers for the treatment of complex tibial fractures and infected nonunions with segmental bone loss. J Orthop Surg (Hong Kong).

[23] Borzunov DY, Gorbach EN, Mokhovikov DS, Kolchin SN (2019). Combined bone plasty interventions for rehabilitation of patients with congenital pseudarthrosis of the tibia. Genij Ortopedii.

[24] Peng J, Min L, Xiang Z, Huang F, Tu C, Zhang H (2015). Ilizarov bone transport combined with antibiotic cement spacer for infected tibial nonunion. Int J Clin Exp Med.

[25] Fischgrund J, Paley D, Suter C (1994). Variables affecting time to bone healing during limb lengthening. Clin Orthop Relat Res.

[26] Paley D (1990). Problems, obstacles, and complications of limb lengthening by the Ilizarov technique. Clin Orthop Relat Res.

[27] Collipal E, Vargas R, Parra X, Silva H, Del Sol M (2007). Diaphyseal nutrient foramina in the femur, tibia and fibula bones/Foramenes nutricios diafisarios de los huesos femur, tibia y fibula. Int J Morphol.

[28] Azi ML, Teixeira AAA, Cotias RB, Joeris A, Kfuri M (2019). Induced-Membrane Technique in the Management of Posttraumatic Bone Defects [published correction appears in JBJS Essent Surg Tech. 2020 Jun 02;10(2):e0099ER]. JBJS Essent Surg Tech.

[29] Selhi HS, Mahindra P, Yamin M, Jain D, De Long WG, Singh J (2012). Outcome in patients with an infected nonunion of the long bones treated with a reinforced antibiotic bone cement rod. J Orthop Trauma.

[30] Apard T, Bigorre N, Cronier P, Duteille F, Bizot P, Massin P (2010). Two-stage reconstruction of post-traumatic segmental tibia bone loss with nailing. Orthop Traumatol Surg Res.

[31] Wang X, Wei F, Luo F, Huang K, Xie Z (2015). Induction of granulation tissue for the secretion of growth factors and the promotion of bone defect repair. J Orthop Surg Res.

[32] Wu M, Chen G, Li YP (2016). TGF-β and BMP signaling in osteoblast, skeletal development, and bone formation, homeostasis and disease. Bone Res.

[33] Carvalho RS, Einhorn TA, Lehmann W, Edgar C, Al-Yamani A, Apazidis A (2004). The role of angiogenesis in a murine tibial model of distraction osteogenesis. Bone..

[34] Fang TD, Salim A, Xia W, Nacamuli RP, Guccione S, Song HM (2005). Angiogenesis is required for successful bone induction during distraction osteogenesis. J Bone Miner Res.

[35] Giotakis N, Narayan B, Nayagam S (2007). Distraction osteogenesis and nonunion of the docking site: is there an ideal treatment option?. Injury..

